# Genome Sequences of *Ralstonia insidiosa* Type Strain ATCC 49129 and Strain FC1138, a Strong Biofilm Producer Isolated from a Fresh-Cut Produce-Processing Plant

**DOI:** 10.1128/genomeA.00847-16

**Published:** 2016-08-18

**Authors:** Yunfeng Xu, Attila Nagy, Xianghe Yan, Bradd J. Haley, Seon Woo Kim, Nancy T. Liu, Xiangwu Nou

**Affiliations:** a College of Food Science and Engineering, Northwest A&F University, Yangling, China; b Environmental Microbial and Food Safety Laboratory, USDA Agricultural Research Service, Beltsville, Maryland, USA

## Abstract

*Ralstonia insidiosa* is an opportunistic pathogen and a strong biofilm producer. Here, we present the complete genome sequences of *R. insidiosa* FC1138 and ATCC 49129. Both strains have two circular chromosomes of approximately 3.9 and 1.9 Mb and a 50-kb plasmid. ATCC 49129 also possesses a megaplasmid of approximately 318 kb.

## GENOME ANNOUNCEMENT

*Ralstonia insidiosa* is a Gram-negative, nonfermentative, aerobic bacillus, along with the closely related better-known species *Ralstonia pickettii*, widely present in aqueous environments, including municipal water and medical water purification systems (1–3). They are reported to be well adapted to survive under low-nutrient conditions and to form biofilms (4, 5). *Ralstonia insidiosa* FC1138 was isolated from a fresh-cut produce-processing plant and determined to be a strong biofilm producer (6) that can promote biofilm formation by *Escherichia coli* O157:H7 (7) and other foodborne pathogens in dual-species biofilms. Currently, only eight fully assembled genomes of *Ralstonia* are available in the NCBI database (http://www.ncbi.nlm.nih.gov/genome/), which do not include any from *R. insidiosa*.

In this study, we present the complete and annotated genome sequences of *R. insidiosa* FC1138 and type strain ATCC 49129. For FC1138, high-quality genomic DNA was extracted from 100 ml of bacterial culture using the MagAttract high-molecular-weight (HMW) DNA minikit (Qiagen, Louisville, KY), according to the manufacturer’s recommendations. The genome was sequenced using a PacBio platform at the Institute for Genome Sciences (IGS), University of Maryland, Baltimore, MD. The IGS Annotation Engine was used for structural and functional annotation of the sequences (http://ae.igs.umaryland.edu/cgi/index.cgi, reference 21677861). The Manatee software package was used to edit and view the annotations (http://manatee.sourceforge.net). ATCC 49129 was sequenced using an Illumina NextSeq platform in our laboratory. *De novo* assembly of clean ATCC 49129 sequenced reads was performed by CLC Workbench 8.5 (Qiagen). The newly assembled open contigs were assembled into circular replicons in reference to the closely related strain FC1138 genome sequences for sequence mapping/alignment using Sequencher version 5.2.2. Annotation was added by the NCBI Prokaryotic Genome Annotation Pipeline (https://www.ncbi.nlm.nih.gov/genome/annotation_prok/).

The genome of FC1138 (5,987,762 bp, 63.6% G+C content) is composed of a primary chromosome (3,968,071 bp), a secondary chromosome (1,968,926 bp), and a large plasmid (50,765 bp). It contains 5,820 protein-coding sequences (CDSs), 54 tRNA, and nine rRNA coding genes. The ATCC 49129 genome (6,177,004 bp, 63.2% G+C content) is similar in composition, including a primary chromosome (3,921,238 bp), a secondary chromosome (1,887,060 bp), and a large plasmid (50,770 bp). In addition, ATCC 49129 possesses a megaplasmid (317,936 bp) that is absent in FC1138. ATCC 49129 encodes 5,731 proteins, 51 tRNAs, and nine rRNAs.

### Accession number(s).

The genome sequences of *R. insidiosa* FC1138 and ATCC 49129 have been deposited in GenBank (http://www.ncbi.nlm.nih.gov/genbank/) under the accession numbers CP012605, CP012606, and CP012607, corresponding to the FC1138 primary chromosome, secondary chromosome, and 50-kb plasmid; and CP016022, CP016023, CP016024, and CP016025, corresponding to the ATCC 49129 primary chromosome, secondary chromosome, 318-kb megaplasmid, and 50-kb plasmid.
